# The Utility of Lipidomic Analysis in Colorectal Cancer Diagnosis and Prognosis—A Systematic Review of Recent Literature

**DOI:** 10.3390/ijms25147722

**Published:** 2024-07-14

**Authors:** Jakub Klekowski, Mariusz Chabowski, Małgorzata Krzystek-Korpacka, Mariusz Fleszar

**Affiliations:** 1 Department of Nursing and Obstetrics, Division of Anesthesiological and Surgical Nursing, Faculty of Health Science, Wroclaw Medical University, 50-367 Wroclaw, Poland; klekowski.jakub@gmail.com; 2 Department of Surgery, 4th Military Clinical Hospital, 50-981 Wroclaw, Poland; 3 Department of Clinical Surgical Sciences, Faculty of Medicine, Wroclaw University of Science and Technology, 50-556 Wroclaw, Poland; 4 Department of Biochemistry and Immunochemistry, Wroclaw Medical University, 50-368 Wroclaw, Poland; malgorzata.krzystek-korpacka@umw.edu.pl (M.K.-K.); mariusz.fleszar@umw.edu.pl (M.F.); 5 Omics Research Center, Wroclaw Medical University, 50-368 Wroclaw, Poland

**Keywords:** colorectal cancer, lipidomics, diagnosis, prognosis, biomarkers, mass spectrometry

## Abstract

Colorectal cancer (CRC) is among the most prevalent and lethal malignancies. Lipidomic investigations have revealed numerous disruptions in lipid profiles across various cancers. Studies on CRC exhibit potential for identifying novel diagnostic or prognostic indicators through lipidomic signatures. This review examines recent literature regarding lipidomic markers for CRC. PubMed database was searched for eligible articles concerning lipidomic biomarkers of CRC. After selection, 36 articles were included in the review. Several studies endeavor to establish sets of lipid biomarkers that demonstrate promising potential to diagnose CRC based on blood samples. Phosphatidylcholine, phosphatidylethanolamine, ceramides, and triacylglycerols (TAGs) appear to offer the highest diagnostic accuracy. In tissues, lysophospholipids, ceramides, and TAGs were among the most altered lipids, while unsaturated fatty acids also emerged as potential biomarkers. In-depth analysis requires both cell culture and animal studies. CRC involves multiple lipid metabolism alterations. Although numerous lipid species have been suggested as potential diagnostic markers, the establishment of standardized methods and the conduct of large-scale studies are necessary to facilitate their clinical application.

## 1. Introduction

Colorectal cancer (CRC) ranks as the third most prevalent malignant neoplasm globally and is the second leading cause of cancer-related mortality. The socioeconomic impact of the CRC is steadily rising, particularly in developed nations, largely attributed to lifestyle factors as primary risk determinants for its development. In 2020, there were an estimated 1.88 billion new cases of CRC diagnosed worldwide. Although CRC typically affects adults aged 60 and above, there is a growing incidence observed among individuals under 50 years of age [1,2,3]. CRC in its early stages can be effectively treated with surgery and sometimes with neoadjuvant or adjuvant oncological therapy. However, in cases of metastatic disease, the chances of survival significantly decline [4].

In the vast majority of cases, CRC evolves within polyps, initially precancerous, eventually developing dysplasia and cancer as a result of accumulated genetic changes. Carcinogenesis for CRC generally takes 10–15 years. It is estimated that only 10% of the most dysplastic adenomas could transform into CRC. The diagnosis of CRC is based on colonoscopy findings with biopsy specimens and histopathological evaluation. There are a handful of promising biomarkers, such as BRAF, KRAS, or NRAS mutations, the CpG island methylator phenotype (CIMP), or microsatellite instability (MSI)—most of which are already utilized. Based on multidimensional biological analysis, the consensus molecular subtypes (CMS) of CRC were established, and four main types were distinguished, which could potentially be a new molecular classification for CRC, allowing a more precise approach to treatment. Although many studies have been conducted to find new prognostic and predictive factors, few have found their place in daily clinical practice. Novel diagnostic measures allowing non-invasive examination are being developed, yet neither has proved efficient enough to be used as a common test [4,5,6,7].

Lipids constitute the main component of the cellular membrane, are the substance of energy storage, play important roles as hormones and signaling molecules, and regulate various metabolic processes [8]. Current consensus classifies lipids into eight main categories: (1) fatty acyls (FA), (2) glycerolipids (GL), (3) glycerophospholipids (GP), (4) sphingolipids (SP), (5) sterol lipids (ST), (6) prenol lipids (PR), (7) saccharolipids (SL), and (8) polyketides (PK). The categories are further divided into classes and subclasses, for example, eicosanoids or fatty acids and conjugates are classes of FA [8,9]. Disturbance in lipid metabolism in cancer has been proven in numerous studies. Cancer cells tend to synthesize more fatty acids despite having a sufficient supply from exogenous sources. Fatty acid synthase expression was found to be increased in lung, breast, and prostate cancers. Rapid cell growth and multiplying—common for most cancers—trigger increased demand for lipids to form new cell membranes and compartments; however, as a result, cancer cells’ membranes generally have altered lipid profiles. Some studies suggest that lipids might be a favorable energy source for a cancerous cell. In addition, lipids exert their function as signaling molecules by regulating metabolic pathways, like the PI3K/AKT signaling pathway. An interesting aspect of lipids’ activity is their influence on tumor immunity, since many lipids like eicosanoids have immunomodulating properties, thereby influencing the tumor microenvironment by recruiting immune cells [8]. Some lipids, such as omega-3 unsaturated FAs, are linked to the reduction in prostaglandin E_2_ (PGE_2_) levels in CRC by modulating cyclooxygenase metabolism. Notably, PGE_2_, a product of FA metabolism, is associated with cell proliferation, migration, and invasion while also promoting an immune-suppressive tumor microenvironment that benefits tumor growth. Additionally, omega-3 FAs are associated with downregulating other CRC-promoting signaling pathways, including the Wnt/ß-catenin pathway, the MAPK/ERK pathway, and the PI3K/PTEN pathway [10,11,12,13,14,15].

Omic studies enable a comprehensive evaluation of a sample’s molecular and metabolite composition. Lipidomics, a branch of omics, focuses on profiling lipids in an untargeted, semi-targeted, or targeted manner. Currently, lipidomics is one of the fastest-growing research fields, as evidenced by the number of publications in recent years. Liquid chromatography (LC) coupled with mass spectrometry (MS) is the most extensively used method for lipidomic studies. Various ionization sources and mass analyzers offer multiple methods of sample analysis, including shotgun lipidomics with direct infusion for analyzing individual lipid species [8,16,17,18].

Monitoring metabolic disorders is currently one of the most rapidly developing pathways in modern diagnostics. Lipidomics, being a relatively young field of research, is gaining increasing recognition in personalized medicine, allowing for highly specific and sensitive monitoring of lipidomic changes in disease states. In a diagnostic context, lipidomics aims to understand the complete composition of lipids (lipidome), which can describe the physiological state, presence or absence, and extent of disturbances at the level of the cell, tissue, organ, and entire organism. Two main research strategies are employed in lipidomic analyses: (1) targeted analysis, which aims to determine specific metabolites involved in processes occurring in well-defined biochemical pathways or groups of compounds characterized by similar physicochemical properties; (2) untargeted analysis, which involves analyzing as many compounds as possible with diverse physicochemical properties participating in various biochemical pathways. The choice of the appropriate approach depends on the researcher’s objective. Untargeted analysis provides a holistic view of changes occurring in the cell, tissue, or whole organism. This strategy allows for monitoring changes in the pool of hundreds or thousands of lipids to identify compounds with the highest diagnostic potential. On the other hand, targeted analysis enables the study of pathological changes based on quantitative relationships between all statistically significant lipids involved in specific biochemical transformations. This helps characterize known or identify new biomarkers for selected diseases.

Currently, two main analytical tools are used in lipidomic studies: (1) nuclear magnetic resonance spectroscopy (NMR), which is characterized by ease of sample preparation and the fact that samples are not destroyed during analysis. However, in the case of complex biological matrices, the sensitivity and complexity of the obtained spectra are low, significantly limiting the possibility of quantitative analysis. This technique is mainly used in untargeted analysis; (2) mass spectrometry (MS), which involves measuring the mass-to-charge ratio (m/z) of ions of the analyzed substances present in the gas phase. Mass spectrometry is highly specific and sensitive, requiring appropriate sample preparation, but when coupled with a suitable separation technique (liquid chromatography (LC), gas chromatography (GC), or capillary electrophoresis (CE)), it allows for the characterization of many groups of compounds and their quantitative analysis at very low levels, even in such complex biological matrices as serum, plasma, urine, tissues, or cell lines. Due to its capabilities, MS is the most commonly used technique in lipidomic studies [19]. Currently, it is most often coupled with LC (LC-MS) due to its versatility, with GC (GC-MS) and CE (CE-MS) as complementary techniques. The most effective sample preparation technique is still liquid–liquid extraction, using methods described almost 80 years ago by Folch et al. [20] and Bligh-Dyer [21], utilizing mixtures of chloroform with methanol in ratios of 2:1 *v*:*v* and 1:2 *v*:*v*, respectively, and the MTBE extraction introduced by Matyash et al. in 2008 [22]. In analyses targeting specific lipid classes, solid-phase extraction (SPE) is also used.

Due to the extraordinary complexity of the lipidome, sophisticated tools are needed to comprehensively understand the interplay within the whole system. Extracting differentiating markers from lipidomic analysis and combining them with clinical, pathological, and laboratory information brings exciting new possibilities for discovering novel prognostic or diagnostic factors. In CRC, lipid biomarkers could prove useful in new tumor phenotyping. Some mechanisms involving lipid metabolism reveal pathways and mechanisms responsible for the pathogenesis, growth, and proliferation of tumors. Furthermore, some pathways could also be potential targets for cancer therapy [8,18,23,24,25,26].

This review aims to gather recent literature findings in CRC lipidomics and identify biomarkers with the potential to provide good diagnostic or prognostic quality.

## 2. Methods

**Figure 1:**
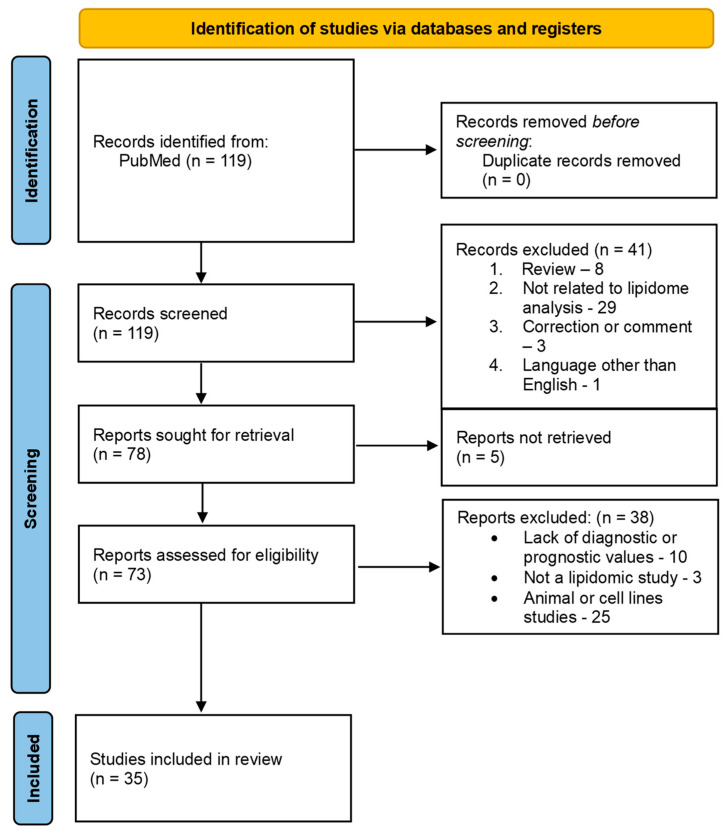
PRISMA flowchart of the literature review process.

We have conducted literature research in the PubMed database using the following combination of keywords: “lipidomics colorectal cancer” within a limited period between 2018 and 2023. A total of 119 records were found. After screening, 78 articles were selected for further evaluation. For the review, original manuscripts concerning lipidomic studies on CRC patients’ derived biological material with specific groups of lipids hallmarked as potential biomarkers were considered. Papers with insufficient information and lacking data, as given above, were excluded. Eventually, 36 papers were included in the results section. The eligible studies were stratified according to the type of biological material studied: blood samples and tissue samples. The detailed research and exclusion process is presented in the PRISMA flowchart (Figure 1).

## 3. Results

### 3.1. Lipidomic Analysis in Blood Samples

Blood is a perfect material for early disease detection, including cancers and other conditions, because it is easy to sample and test quickly. Therefore, it is understandable that a lot of research is focused on finding new indicators in the blood that can signal the presence of various diseases, including cancer.

There are 21 articles included in this section of the review. Several of them involve untargeted lipidomic analysis. The untargeted approach allows for a comprehensive evaluation of the lipidome, including polar membrane lipids like glycerophospholipids or sphingolipids, as well as non-membrane lipids and lipid metabolites. On the other hand, targeted analysis provides detailed information about specific classes and groups of lipids, which is used for meticulous biomarker studies.

Regarding the articles featured in Table 1, it is essential to note that the studies exhibit significant heterogeneity in terms of studied groups and methodology, precluding the synthesis of a meta-analysis. Consequently, the overview of the available literature offers a broad perspective, indicating merely tentative patterns and trends in lipidome alterations.

Membrane lipids, especially phosphatidylethanolamines (PEs) and phosphatidylcholines (PCs), are recurring themes in the analyzed articles. PEs generally follow a pattern of decreasing in the blood of CRC patients [27,28,29]. In one of the articles, this observation is reversed, as PE (18:2/16:0) was increased in the cancer group, but the *p*-value was not significant [30]. Other results come from the lipidome analysis of plasma exosomes. PEs were markedly more abundant in non-metastatic CRC patients than in healthy controls, although this relationship diminished when compared to the metastatic group. More importantly, patients with metastasis showed lower levels of PEs than patients without metastasis [31]. A significant increase in PE 38:4 was found, while PE 34:1 was decreased (significantly in hereditary syndrome cancer) [32].

PCs are major constituents of cell membranes and thus are one of the most commonly reported lipids in lipidomic analysis. It is difficult to establish a general pattern that would apply to PCs as a class of lipids. Theoretically, given the vast cell turnover in CRC, the concentration of PCs would be expected to increase. Conversely, due to the increased expression of phospholipase A2 reported in some cancers, the levels of PCs might be lower [33]. Several articles report decreased levels of PCs, especially PC 36:2, 36:1, 38:4, 36:6, and 22:1/12:3 [28,33,34,35]. On the other hand, there are also studies showing a trend of increased concentrations of PCs in blood samples—such as PC 32:3, 37:7, 18:1/16:0, 18:1/16:1, 18:0/20:5, 18:0/22:6, and 16:0/22:6 [30,33,35]. Circulating exosome analysis also reports variations in PC levels. One of the articles points out decreased PC 34:1 in CRC groups, especially in hereditary CRC syndromes; however, in another study, PC 34:1 shows increased levels in CRC patients without metastasis compared to controls, although this changes when comparing non-metastatic patients with metastatic patients, which tend to have lower levels. PC 34:2, 36:4, 36:2, and 38:4 showed an increasing tendency; however, not all reached statistical significance [31,32]. The reports from studies concerning precancerous adenomas are inconsistent. In two separate articles PC 31:2, PC 37:7, and PC 44:5, there were conflicting trends. Other PCs like PC 35:6, 36:3, and 41:8 were found to be increased in patients with adenomas, while PC 30:1, 18:0, and 41:9 were decreased [33,36]. Another study exploring lipidomic profiles explaining the adenoma–carcinoma pathway showed a negative correlation with increasing malignancy [37]. PC 22:1/12:3 was reported to be an unfavorable prognostic factor [35].

A greater consistency than in PC is observed in LPCs. Reports show generally decreased levels of LPCs in blood samples of the studied groups when compared to healthy controls, especially prominent are LPC 17:0 and 19:0 [35,36,37]. A study exploring the exosome’s lipids showed less marked differences. Only in adenomas, LPC 18:1 and 20:4 were increased, while LPC 16:0 was decreased. In other groups, the differences were not significant [32]. In targeted lipidomics, four LPCs were included in a diagnostic model (AUC = 0.863), differentiating CRC from healthy controls [38]. In one of the studies, patients with higher levels of LPC 17:0 had a decreased survival rate [35].

Ceramides exhibited altered levels in CRC patients, although very few of them reappear across reported studies, which interrupts the possibility of comprehensive analysis. The results of the studies show discrepant trends with increased or decreased levels of ceramides. Among the lowered ceramides, Cer (d18:1/23:0), (m19:0/21:2), (m19:0/22:2), (d18:1/24:1), and CerP (d15:0/22:0+O) were noted. On the other hand, several other ceramides were found to be significantly upregulated—Cer (t18:0/19:0), (d18:3/20:1), or GlcCer (d14:2/16:0) [28,30,35]. In one study of plasma exosomes, ceramides were almost uniformly increased in CRC compared to controls; however, not all reached statistical significance. Notably, when comparing metastatic and non-metastatic patients, the ceramide levels were lower in metastatic patients—especially HexCer (d18:1/24:0) and (d18:1/24:1)—which were statistically significant [31]. In another exosomes study, ceramides showed no statistically significant differences across healthy controls and patients with adenomas and CRC [32].

According to the available data, most TAGs are downregulated in CRC when compared to healthy controls. The same observation was made in patients with advanced adenomas. Interestingly, Liu et al. found that TAGs are upregulated in more advanced stages of CRC (III/IV vs. I/II). It was also proposed that TAG (11:0-18:0-18:0) and TAG (18:0-18:0-18:1) could be prognostic factors since patients with higher levels of those lipids had better survival rates [35,36,39,40].

**Table 1:** Blood lipid biomarkers.

Material	Analysis	Method	Featured Lipids	Results and Conclusions	Reference
Serum of 66 CRC cases 66 controls	Untargeted	LC-MS, MS/MS	9 selected metabolites associated with case–control status: 5 unknown classes, one identified as ULCFA 468; the remaining three were likely a fatty acid, a ULCFA, a ceramide	4 metabolites were associated as causal features (3 unknown + possible ceramide) with a correct classification rate of 72% Other 4 features (ULCFA, fatty acid, and unknown) were associated with cancer progression and could be diagnostic markers.	[41]
Plasma of 40 CRC patients	Untargeted	LC-MS, MS/MS	CE (20:4) FAHFA 27:1 (9:0/18:1) TAG 40:0 (12:0/12:0/16:0) TAG 42:0 (12:0/14:0/16:0) TAG 44:0 (14:0/14:0/16:0) TAG 46:0 (14:0/16:0/16:0) TAG 48:0 (16:0/16:0/16:0) TAG 54:0 (16:0/18:0/20:0)	8 lipids were selected as biomarkers for a statistical model discriminating CRC stages I–II from stages III–IV. All lipids except FAHFA were increased in the higher stages of CRC. The area under the curve (AUC) was 89.8%, with 85% sensitivity and 80% specificity.	[39]
Plasma of 51 stage I/II CRC patients and 52 healthy controls	Untargeted and targeted	LC-MS, MS/MS	PE (18:2/16:1), PE (P-18:2/18:2), PE (P-18:1/18:2), PE (P-18:1/22:5), PE (O-18:0/16:0), FFA (20:5), FFA (22:4), FFA (20:0), FAHFA (16:0/18:2), PA (20:0/18:2) and PA (18:0/18:2)	The featured lipids were used in a model distinguishing healthy from disease, with an AUC of 0.981. The levels of FFAs were higher in CRC patients, while PAs and PEPs were lower.	[27]
Serum of 25 CRC patients and 16 controls	Untargeted	LC-MS	Cer (t18:0/19:0), Cer (d18:3/20:1), Cer (d18:0/13:0), GlcCer (d14:2/16:0) PC (P-18:0/20:5) PC (18:1/16:0) PC (18:1(11Z)/16:1(9Z)) PC (18:2(9Z,12Z)/0:0) PC (16:0/16:1) PC (18:0/20:2(5Z,11Z)) PC (18:0/20:5(5Z,8Z,11Z,14Z,17Z)) PA (22:5(7Z,10Z,13Z,16Z,19Z)/0:0) PA (24:4/0:0) PE (18:2/16:0) PG (14:1/14:1) Linoleyl stearate Stearyl palmitate CE 20:3, CE 20:1, CE 22:3 Retinol oleate 9-Hexadecenoylcholine Tetracosapentaenoic acid (24:5n-3) DG (16:0/18:0/0:0) MG (18:2(9Z,12Z)/0:0/0:0) [rac]	25 molecules were identified as potential biomarkers based on AUC (>0.75 and *p*-value). Choline-dependent phospholipids, ceramides, and different esters (of fatty acids or cholesterol) could be considered as biomarkers. The most prominent lipid was Cer (t18:0/19:0) with AUC = 0.94056. Most lipids were found to be increased, but only 9-hexadecenoylcholine, 20:1 CE, and tetracosapentaenoic acid (24:5n-3) were decreased.	[30]
Plasma of 36 CRC patients and 37 controls	Untargeted	MALDI/TOF-MS	4 sphingolipids 3 glycerophospholipids 1 polyketide	A model with a ROC curve using the featured molecules showed an AUC of 0.87. Three of those lipids were associated with survival.	[42]
Plasma of 16 CRC patients and 20 controls	Untargeted and targeted	LC-MS, MS/MS	PC (36:2) PE (34:1); PE (34:2); PE (36:1); PE (36:2); PE (36:3); PE (36:4); PE (38:3); PE (38:4); PE (38:5); PE (38:6); PE (40:5); PE (40:6); PE (16:1p/22:6); PE (18:0p/20:4); PE (18:1p/20:4); PE (18:1p/20:2); PE (18:1p/22:4); PE (16:0p/20:4); PE (18:1p/22:5) Cer (d18:1/24:1) PG (18:1/18:1) PI (18:1/18:0)	The listed lipids showed at least 2-fold significant changes compared to controls—all showed significant decreases. In ROC analysis, (AUC > 0.8) PC (36:2); PE (36:1); PE (38:4); PE (38:6); PE (16:0p/20:4); PE (18:0p/20:4); PE (18:1p/18:1); PE (18:1p/22:4) were included in the model. PC (36:2) was specific for CRC, while other lipids were common in other studied cancers.	[28]
Plasma of 25 CRC patients and 50 controls	Targeted	LC-MS, MS/MS	PC (36:2); PC (36:1); PC (38:4); PC (38:6) PEP (P-16:0/20:4); PEP (P-18:0/20:4); PEP (P-18:1/18:1); PEP (P-18:1/22:4)	All reported lipids were decreased in CRC with strong statistical significance. AUC values ranged from 0.757 to 1.000.	[34]
Plasma of 96 CRC patients (including 18 patients with liver metastasis) and 29 cancer-free individuals	Untargeted	LC-MS, MS/MS	PS (40:1) Sphingosine LPE (18:1) CE (22:6); CE (18:3) PS (18:0/23:3) TAG (56:9) FFA (16:1) PS (P-32:1)	Multiple linear regression with expectation maximization featured 9 lipids the most accordingly classifying patients in the studied groups. PS (40:1), CE (22:6), PS (18:0/23:3), and TG (56:9) were statistically significant.	[40]
Plasma of 17 CRC patients and 27 non-cancer controls	Untargeted	GC-MS, LC-MS	PE 34:2 PE 34:3 PE 36:4	The featured lipids showed good diagnostic ability (with AUC > 0.95) between CRC and control.	[29]
Plasma of 49 CRC patients and 50 controls	Untargeted	LC-MS, MS/MS	CerP (d15:0/22:0+O) TAG (18:4/18:2/18:2) TAG (18:3/18:3/18:3) TAG (18:0/18:0/18:1) TAG (20:3e/18:4/18:4) TAG (14:0/18:2/18:2) TAG (20:4+O/16:0/16:0) AC (16:1) Cer (d18:1/23:0) LPC (20:2)	In the analysis, 10 lipids were found to be upregulated and 31 were downregulated in CRC. 10 lipids were selected as the best characterizing CRC form controls; however, only CerP (d15:0_22:0+O) showed good accuracy above AUC > 0.9. Four lipids had significant survival prognostic values: TAG (11:0_18:0_18:0) (HR: 0.34), TAG (18:0_18:0_18:1) (HR: 0.34), PC (22:1_12:3) (HR: 2.22), LPC (17:0) (HR: 3.16).	[35]
Serum samples of 62 CRC patients, 31 patients with non-malignant adenomas, and 81 controls	Targeted	MS/MS, GC-MS	Several PCs and LPCs are included in the metabolomic model	Based on GC-MS/MS analysis models differentiating CRC and adenomas from healthy controls were established. Several PCs and LPCs were included in the CRC/control model, while in the adenoma/control model, PC 40:2 and LPC 17:0 were selected.	[43]
Plasma of 40 CRC patients, 12 patients with adenomas, and 32 controls	Untargeted	MALDI-TOF/MS	3 polyketides, 1 glycerolphospholipid, 4 fatty acids	There were no differences between controls and adenomas. CRC patients differed from controls in 8 lipids. Concentrations of 6 were lower in CRC patients, while 2 lipids were upregulated—which were identified as an endocannabinoid and a hydroxy fatty acid.	[44]
Serum of 46 patients with advanced adenoma and 50 controls	Untargeted	LC-MS	PC 35:6e, PC 44:5, PC 31:2, PC 37:7, PC 42:9, PC 18:0e TAG 57:1 LPC 18:0, LPC 17:0 Methyl palmitate Palmitic acid Docosanamide	12 differential lipids showed good diagnostic performance (AUC > 0.90). Out of those PC 44:5 and PC 35:6e had the highest accuracy. In the adenoma group levels of LPCs, PC 18:0e, PC 42:9, and TAG 57:1 were decreased, and others were increased.	[36]
Serum of 50 patients with colorectal adenoma and 50 controls	Untargeted	LC-MS	4-dodecylbenzenesulfonic acid PC 44:5, PC 30:1, PC 31:2, PC 41:8, PC 37:7, PC 36:3, PC 21:4 Palmitoyl ethanolamide Methyl palmitate Palmitic acid 2-arachidonoyl glycerol	12 lipids were selected with significant accuracy in ROC curves with AUC > 0.9. PC 41:8, PC 36:3, palmitoyl ethanolamide, methyl palmitate, and palmitic acid were significantly up-regulated in the adenoma group, while others were down-regulated.	[45]
Serum of 66 CRC patients, 76 patients with advanced adenomas and 93 controls	Targeted	LC-MS/MS FIA-MS/MS	PCs LPCs ACs SMs	PC 34:4 was found to be the best discriminating factor between CRC and control (AUC of 90.7%) PC aa C36:5 is the most accurately discriminated adenomas and CRC (AUC = 83.1%). Lipids overall had the highest power to distinguish groups.	[37]
Serum samples of 20 CRC patients, 23 patients with adenomas, and 21 controls	Targeted	HR-TOF/MS	PUFAs SFA	Omega-3 PUFAs were found to be downregulated in CRC compared to adenomas and controls. Omega-6 PUFAs and c18 SFA showed a reverse trend.	[46]
Serum samples of 50 CRC patients and 50 patients with adenoma	Untargeted	LC-MS	Docosanamide PC 36:1e, PC 37:7, PC 32:3 Triheptanoin SM d36:0, SM d36:1	The featured lipids showed the best performance in discriminating CRC from adenoma. Docosanamide, PC 37:7, PC 32:3, and triheptanoin were downregulated in the adenoma group.	[33]
Plasma exosomes of 12 patients: metastatic and non-metastatic CRC, and healthy controls	Targeted	LC-MS/MS	PC 36:4, PC 36:5, PC 34:1, PC 34:2 PE 38:4, PE 38:5, PE 36:2, PE 34:2 PE(P-18:0/20:4), PE(P-16:0/20:4) PI 36:1, PI 36:2, PI 34:1 PS 18:0/22:5, PS 18:0/22:6, PS 18:0/20:3, PS 18:0/20:4, PS 18:0/18:1, PS 18:0/18:2, PS 16:0/18:1, PS 16:0/18:2 SM d18:1/24:1, SM d18:1/16:0 Cer d18:1/24:0, Cer d18:1/24:1, Cer d18:1/23:0, Cer d18:1/22:0, Cer d18:1/16:0, Cer d18:2/16:0 HexCer d18:1/24:0, HexCer d18:1/24:1, HexCer d18:1/16:0, HexCer d18:2/16:0	PC 34:1, PE 36:2, SM d18:1/16:0, HexCer d18:1/24:0, and HexCer d18:1/24:1 were suggested as biomarkers for non-metastatic cancer when compared to controls. On the other hand, PE 34:2, PE 36:2, PE(P-16:0/20:4), and Cer d18:1/24:1 best characterized the metastatic patients. The findings were supported by a cell lines study.	[31]
Plasma exosomes of 28 patients with CRC (including 9 patients with hereditary syndromes), 21 patients with hyperplastic and adenomatous polyps, and 13 healthy controls	Targeted	FIA-MS/MS	PC 34:2, PC 34:1, PC 36:4, PC 36:2, PC 36:1, PC 38:4 PE 34:1, PE 36:2, PE 36:1, PE 38:6, PE 38:5, PE 38:4, PE 42:7 PI 32:0, PI 34:1, PI 36:2, PI 36:1, PI 36:0, PI 38:4 LPC 16:0, LPC 18:1, LPC 18:0, LPC 20:4 SM 34:1, SM 36:1, SM 40:1, SM 42:2, SM 42:1 Cer 18:1/16:0, Cer 18:1/22:0, Cer 18:1/23:0, Cer 18:1/24:1, Cer 18:1/24:0	PC 34:1, PE 34:1, and PI 34:1 were decreased in pathologic groups, while PC 38:4, PE 38:4, and PC 38:4 showed increased levels. A total 34:1/38:4 ratio had 54.6% sensitivity in classifying colorectal lesions.	[32]
Plasma samples CRC: 9 pilot, 28 validation Control: pilot 15, 23 validation	Targeted	HR-MS	VLCDCAs	VLCDCA 28:4 was decreased in the plasma of CRC patients.	[47]
Plasma samples of 21 CRC patients and 38 controls	Targeted	LC-MS, MS/MS	LPC 17:0; LPC 19:0; LPC 19:1; LPC 19:2	4 LPCs were selected in a model (AUC = 0.863) differentiating CRC from healthy controls.	[38]

ULCFA—ultra-long-chain fatty acid; VLCDCA—very-long-chain dicarboxylic acid; TAG—triacylglyceride; DG—diacylglycerol; MG—monoacylglycerol; Cer—ceramide; GlcCer—glucosylceramide; HexCer—hexosylceramide; FAHFA—fatty acyl esters of hydroxy fatty acid; FFA—fatty acid; PE—phosphatidylethanolamine; PA—diacylglycerophosphate; PEP—ethanolamine plasmalogen; PG—phosphatidylglycerol; PI—phosphatidylinositol; PC—phosphatidylcholine; LPC—lysophosphatidylcholine; PS—phosphatidylserine; LPE—lysophosphatidylethanolamine; CE—cholesteryl ester; CerP—ceramide phosphate; AC—acylcarnitine; SM—sphingomyelin; SFA—saturated fatty acid.

### 3.2. Lipidomic Analysis in Human Tissue Samples

This section comprises 14 items, all of which are summarized in Table 2. Studies utilizing clinical tissue samples were fewer in number compared to those involving blood samples. It is evident that there is considerable variation in the research regarding the exploration of different lipids, methodologies employed, patient populations, and, most importantly, the outcomes.

An inflammatory background plays a crucial role in the development and progression of CRC. The tumor microenvironment (TME) relies heavily on cytokine activity, which regulates metabolism across numerous pathways. Lipids are essential for cytokine production and serve as important signaling molecules in inflammation regulation. In this context, it is imperative to consider fatty acid (FA) metabolism, particularly polyunsaturated fatty acids (PUFAs), such as eicosanoids produced from omega-6 and omega-3 FAs-like AA, EPA, DHA, and GLA. Eicosanoids synthesized from AA exhibit proinflammatory activity, while those derived from EPA exert anti-inflammatory functions. Therefore, the discovered dysregulation of the AA/EPA ratio, with its increase in metastatic CRC, is a promising candidate for a biomarker. It was suggested that endocannabinoids’ (eCB) activity could be partially responsible for the observed disruptions. EPA levels were found to be lowered, while GLA was increased in metastatic CRC. Therefore, the PUFA ratio omega-6/omega-3 was accordingly higher in metastatic CRC. Since AA and GLA are both omega-6 PUFAs, this relationship underscores the increased inflammatory activity in metastatic tumors [48,49]. In another study, CRC tissue was found to be enriched in AA, but increased levels of omega-3 PUFAs were also detected. However, the PUFA ratio was not analyzed, making it difficult to compare these results [50]. Other authors report increased MUFAs and PUFAs, as well as upregulated longer-chain and more unsaturated FAs in cancer tissues. One research stands out in this comparison, reporting no differences in FA levels between cancer and control [51,52,53].

TAGs in blood samples were predominantly observed to be reduced in CRC patients. A similar finding was noted by certain authors in tissue studies, where TAG levels were lower in cancer samples [50,54]. Conversely, in one study, TAGs showed a tendency to be upregulated in the studied cohorts. Especially TAGs containing PUFAs in their chains were elevated. The authors proposed that a specific signature of TAGs could be used as a prognostic marker for survival. Notably, the increased TAGs and PUFAs were determined to be solely of dietary origin [55].

The examinations in this section explored various lysophospholipids (LPLs). With some exceptions, the literature generally suggests increased levels of LPLs in tumor tissues. Three studies showed an elevation in LPC, while one study reported the opposite trend. A comprehensive analysis in one study revealed that unsaturated chains predominantly constitute the increased LPCs. Elevated levels of LPI and LPS were observed in tumors. Conversely, LPAs were found to be decreased in CRC [51,53,55,56,57].

Different levels of ceramides were found in CRC tissues. According to one study, tumor samples contained more ceramides with longer chains (C24:0–C26:0) and fewer ceramides with shorter chains (<C22:0). However, some studies found a slightly but significantly higher total amount of ceramides. On the other hand, Guo et al. reported a decrease in ceramide species in CRC, which was linked to the methylation of the enzyme essential for ceramide metabolism [53,55,57].

**Table 2:** Tissue lipid biomarkers.

Material	Analysis	Method	Featured Lipids	Results and Conclusions	Reference
11 samples from CRC and surrounding healthy tissues	Targeted	LC-MS/MS	Lysophospholipids: LPI, LPG, LPS, LPA, LPC, LPE	Total amounts of LPI and LPS were significantly higher in CRC. LPG, LPC, and LPE, although were upregulated in CRC, did not reach statistical significance. LPA levels were found to be lower in CRC. The main increased lipids in tumor tissue were LPI 18:0, LPI 20:4, LPG 18:1, LPG 22:6, LPS 18:0, LPS 18:1, LPS 20:3, LPS 20:4, LPS 22:6. Significantly decreased lipids were LPA 18:1 and LPA 18:2.	[56]
68 samples from CRC and normal mucosa—35 non-metastatic and 33 metastatic patients	Targeted	GC	Arachidonic acid (AA), eicosapentaenoic acid (EPA)	CRC patients with metastases showed a higher AA/EPA ratio compared to CRC patients without metastases, both in non-tumor adjacent mucosa and in tumor tissue (only the ratio between tumors was significant).	[48]
11 stage I and IIA CRC samples and normal mucosa	Targeted	GC-MS, LC-MS	Fatty acid esters of hydroxy fatty acids (FAHFA); 9-hydroxystearic acid (9-HSA)	Tumor tissues contain significantly lower amounts of 9-HSA than normal mucosa.	[58]
25 CRC tissue samples	Untargeted	GC-MS, NMR	TAGs, phospholipids, cholesterol, MUFAs, PUFAs, SFAs	Total lipid content was lower in cancer tissue than in normal tissue; however, only TAGs were found to be lower in cancer, while free cholesterol, phospholipids, PEs, SMs, and PCs were higher. CRC contained significantly fewer MUFAs and oleic acid. On the other hand, CRC tissue showed higher levels of SFAs and n-3 and n-6 PUFAs, as well as 2-fold higher concentrations of EPA, DHA and 2.5-fold higher levels of AA. SFA, stearic acid, was also increased in CRC.	[50]
20 CRC and healthy tissue samples	Untargeted	Shotgun lipidomics	Cholesterol, CE, TG, DG, PC, PE, PS, PI, PG, PA, LPC, LPE, LPI, LPA, Cer, SM	Total lipid content did not differ significantly between tumor and normal tissue. PE, LPI, and Cer were increased in CRC, whereas LPC and LPE were decreased.	[57]
6 CRC, 11 adenomas, and 14 healthy tissue samples	Targeted	LC-MS, MALDI-IMS	Ethanolamine plasmalogens	Ethanolamine plasmalogens were found to influence colonocyte differentiation with differences between colon mucosa layers. PEP 36:4 was significantly increased in CRC when compared to adenoma and healthy tissue. PEP 38:4 and PEP 40:6 were most upregulated in adenomas, while the level in cancer tissue was the lowest.	[59]
32 CRC and healthy tissue samples	Untargeted	LC-MS	Eicosanoids, FFA, LPC, LPE, LPG, LPI, SM, Cer, CE, TAG, AC	The lipidome analysis revealed 131 significantly upregulated and 115 downregulated lipid metabolites. Eight ceramides were significantly decreased in cancer: Cer (d18:1/20:0), Cer (m18:1/20:0), Cer (m18:1/22:0), Cer (m18:1/22:1), Cer (m18:1/24:1), Cer (d18:1/18:0), Cer (d18:1/20:1) and CerP (d18:1/12:0). Among 20 the most dysregulated lipids, ceramides were the only which were downregulated. Other upregulated lipids included 13 FFAs, 2 PCs, a CE, and an LPC.	[53]
22 CRC and healthy tissue samples (isolated EpCAM+ epithelial cells)	Targeted	LC-MS/MS, GC-MS	Phospholipids, lysophospholipids, and fatty acids (FA)	Total levels of FAs were not significantly increased in isolated tumor cells; however, 13 specific FAs were found to be upregulated in cancer. At the same time, total n-3 PUFAs were increased in cancer cells. Total levels of phospholipids and lysophospholipids did not differ significantly between cancer and control. Nevertheless, PC 32:0, 32:1, 36:5, and 38:4 were upregulated in cancer. Furthermore, several species of LPC, LPE, and LPS were also upregulated in cancer cells.	[51]
31 CRC tissue samples (16 with peritoneal metastasis and 15 without metastasis)	Targeted	LC-MS/MS	FFA in cancer-associated fibroblasts (CAFs)	No difference in FFA levels between groups was found in the study.	[52]
45 CRC tissue samples (23 with peritoneal metastasis and 22 without metastasis)	Untargeted	LC-MS/MS	SM, PC, PS, PI, PE (in CAFs)	Total levels of all featured lipid species were upregulated in tumors with metastasis.	[60]
13 rectal cancer tissue samples—before and after neoadjuvant chemo-radiotherapy (CRT)	Untargeted	LC-MS/MS	Phospholipids, oxidized phospholipids, sphingolipids	Several lipid species’ alterations were detected in tissue samples among patients responding and not responding to CRT.	[61]
12 CRC tissue samples and control healthy tissues	Targeted	Reverse phase-UPLC-ESI-MS/MS	Odd-chain FAs	In the odd-chain-FAs lipidomic analysis, TAGs were lowered in tumor tissues compared with the adjacent normal tissues.	[54]
154 CRC and matched healthy tissue samples (divided into cohorts)	Untargeted	ESI-MS/MS, FIA-MS/MS	PC, phosphatidylcholine-ether, LPC, PE, LPE, PEP, PI, PS, SM, Cer, HexCer, free cholesterol and CE, DAG, and TAG	Tumor tissue contained significantly higher proportions of mono- and polyunsaturated LPC (16:1, 18:1, 20:4, and 22:6), SMs with 32–34 carbons, Cer with longer chains (C24:0–C26:0), and TAGs with PUFAs with 56 carbons. On the other hand, SMs with more than 34 carbons were decreased in tumors, as well as shorter-chain ceramides (<C22:0) and TAG species with <53 carbons. Classification scoring based on TAG, SM, and Cer (or without Cer in other cohorts) signatures was able to discriminate tumors from non-diseased tissue AUC > 0.83.	[55]
51 CRC (25 with metastasis) and healthy mucosa tissue samples	Targeted	GC	FAs	Metastatic CRC patients showed significantly lower levels of EPA, but higher GLA. In normal mucosa, a slight increase in the SFA, n-6-PUFA/n-3-PUFA ratio, and GLA levels and a decrease in EPA levels were detected in metastatic CRC patients.	[49]

LPI—lysophosphatidylinositol; LPG—lysophosphatidylglycerol; LPS—lysophosphatidylserine; LPA—lysophosphatidic acid; MUFA—mono-unsaturated fatty acid; PUFA—poly-unsaturated fatty acid; DHA—docosahexaenoic acid; EPA—eicosapentaenoic acid; SFA—saturated fatty acid; GLA—γ-linolenic acid.

## 4. Discussion

### 4.1. Importance of Cell Culture and Animal-Based Studies

Analysis of blood and tissue samples sheds light on the lipidomic changes linked to colorectal cancer. Studies employing tissue samples offer a detailed understanding of the lipid composition within the tumor microenvironment, while those utilizing blood samples reflect systemic changes that could serve as diagnostic or prognostic biomarkers. Though research involving tissue and blood samples provides insightful data, there are obstacles and restrictions associated with them. Tissue studies are often limited by sample availability and heterogeneity. Studies using blood samples are less invasive and provide systemic insights, but they might not be as specific as tissue-based analysis. Additionally, both types of studies exhibit considerable heterogeneity in methodologies, patient populations, and reported outcomes, making it challenging to synthesize findings across studies (Table 3).

Nevertheless, in tissue studies, PCs and LPCs were often found to be dysregulated, with varying trends depending on the study. Additionally, ceramides exhibited diverse patterns. On the other hand, in blood sample studies, PEs and TAGs were commonly found to be dysregulated, with a general trend towards decreased levels in CRC compared to healthy controls. Lysophospholipids, particularly LPCs, are often increased in blood samples from CRC patients, indicating their potential as circulating biomarkers.

In addition to studies involving tissue and blood samples from human subjects, research utilizing animal models and cell lines also provides crucial insights into the lipidomic alterations associated with CRC. They provide valuable mechanistic understanding, identify potential therapeutic targets, and serve as a platform for the preclinical testing of novel interventions. Integrating findings from these diverse research approaches enhances our overall understanding of CRC pathogenesis and facilitates the development of effective diagnostic and therapeutic strategies.

Lipidomic analysis has the potential to reliably classify various cell lines according to their distinct lipid profiles. It is essential to take into account each cell line’s unique lipidome when conducting research using cell lines [62]. Several aspects of the metabolism of MUFAs and PUFAs are better understood because of in vitro studies. WNT signaling in cancer stem cells (CSCs) may be supported by MUFAs synthesized due to the increased activity of stearoyl-CoA desaturase 1 (SCD1). PEs carrying MUFAs are more abundant in CSCs. It has been shown that stem cell maintenance requires a particular lipid content [63]. In another study, DHA decreased MUFA metabolism and enhanced n-3 fatty acid production. Furthermore, the expression of proteins involved in fatty acid synthesis, such as fatty acid synthase, SCD1, and elongase 5, was changed by DHA (and/or butyrate) [64]. In cells overexpressing fatty acid 2-hydroxylase (FA2H), lipidomics analysis showed a buildup of PUFAs, possibly as a result of nutritional deprivation. Moreover, the quiescent and necrotic areas of the spheroid model exhibit elevated PUFA-containing lipid synthesis [65,66]. The aforementioned findings could provide insight into previously reported research on tissues, which noted elevated levels of PUFAs and MUFAs in tumors. This could be attributed to the increasing presence of necrotic cells in growing tumors, resulting from impaired oxygenation and nutrient supply. It is notable that colorectal cancer (CRC) cells exhibit a response to hypoxia through the modulation of ceramide metabolism. Research indicates a decrease in Cer 16:0 levels and an increase in Cer 24:0 and Cer 24:1 levels, indicative of a shift favoring cancer survival and proliferation. Additionally, a decrease in ceramide synthase 4 and 5 levels was observed, coinciding with an augmented rate of cell proliferation. The depletion of these enzymes has been proposed as a potential prognostic factor [67,68].

The lipid composition of cancer cells undergoes alterations in response to chemotherapy. Specifically, treatment with 5-fluorouracil (5-FU) leads to a reduction in levels of AC 4:0, PC 30:0, and PC 32:2 [69]. Moreover, separate research has identified that resistance to 5-FU is correlated with an increase in SM levels and a decrease in Cer levels [70]. Upon treatment with oxaliplatin, there was an observed increase in plasma membrane viscosity, which was associated with reduced PC levels and elevated cholesterol content. Conversely, a separate investigation revealed marked rises in TG and CE species, particularly notable in oxaliplatin-sensitive cells compared to resistant ones. Furthermore, oxaliplatin treatment led to significant elevations in phospholipids and PUFA chains, while TG-containing saturated or monounsaturated fatty acid chains were notably downregulated [71,72,73].

Research suggests the potential to differentiate tumor cells from healthy tissues, as well as transformed cells from non-transformed cells, through lipidomic analysis [74,75]. Gong et al. demonstrated that cancer-associated fibroblasts (CAFs) play a pivotal role in promoting cancer cell migration and growth by accumulating higher levels of fatty acids and phospholipids, which are then taken up by CRC cells subsequent to secretion from CAFs. Fatty acid synthase was identified as the key factor responsible for this process [76]. Cell culture and animal studies also investigate alterations in the lipid composition in response to various substances, including potential therapeutics, as well as in cells with specific mutations [77,78,79,80,81]. Research in murine lipidomics suggests that adding oleic acid to the diet decreases intestinal inflammation and tumor formation in mice. Additionally, the cytochrome P450 monooxygenase pathway, crucial for the in vivo metabolism of linoleic acid (LA), is necessary for the cancer-promoting effects of LA. This is evidenced by the fact that a diet rich in LA does not worsen colon cancer in mice deficient in CYP monooxygenase [82,83].

### 4.2. The Landscape of Lipidomics in CRC

Previously published reviews have demonstrated various dysregulations in lipid metabolism in colorectal cancer (CRC). These studies elucidate the mechanisms underlying disruptions in the lipidome and their potential implications. Salita et al. identified eight hallmark alterations associated with lipid metabolism in CRC: (1) increased cell signaling, (2) increased pro-inflammatory signaling, (3) reduction in pro-resolving inflammatory signaling, (4) disruption of energy homeostasis, (5) dysregulation of lipid raft dynamics, (6) avoiding cell death, (7) reduction in mitochondrial respiration, and (8) dysregulation of membrane homeostasis. Each hallmark was accounted for accordingly. Several lipid species are recurring themes in lipidomic analysis. Ceramides, TGs, LPLs, and PLs are mainly pointed out [24,25,26,84]. However, a consensus on the lipidomic signature of CRC has yet to be established, possibly due to several limitations. Firstly, the studies conducted often exhibit considerable heterogeneity in terms of cancer stage, site, and subtype. Additionally, factors such as diet, malnutrition, medication use (especially those affecting lipid metabolism), and prior treatments (e.g., chemotherapy) are rarely consistently considered or may vary between studies. Furthermore, the sampling methods employed contribute to the heterogeneity of the studies. Moreover, the majority of studies have relatively small sample sizes, typically fewer than 100 subjects, which limits the generalizability of the findings to larger cohorts. Variations in analysis methods, including targeted versus untargeted approaches, further add to the challenges of synthesizing the results. These factors collectively contribute to a situation where there is a substantial body of research in the field, but meta-analysis is challenging.

Despite these challenges, recent literature reflects numerous endeavors aimed at developing diagnostic lipid sets that could function as biomarkers for the early detection and classification of CRC. Notably, in blood samples, statistical models have prominently featured TAGs, phospholipids—particularly PCs and PEs—as well as sphingolipids, with a particular emphasis on ceramides [27,29,30,35,36,37,39,40,42,43,45]. Models of this nature were not commonly developed in tissue-based studies; however, the study by Ecker et al. is notable for presenting a scoring lipid signature capable of distinguishing CRC from healthy tissues [55]. 

Lipidomic studies on CRC have also brought attention to an intriguing topic not covered in this review: the endocannabinoid system (ECS). This extensive modulating network of signaling compounds, along with their receptors, is involved in numerous regulatory pathways. Studies indicate that ECS activity could play a significant role in CRC development and growth. The fact that the main ligands for ECS receptors—arachidonoyl ethanolamine and 2-arachidonoylglycerol—are lipids underscores the relevance of lipidomic studies in this area [85,86].

### 4.3. The Genetic Hallmarks and Omic Studies in the CRC

The genetic profile of cancers has increasingly become foundational for personalized treatment across various neoplasms. Nonetheless, the adoption of comprehensive genetic testing in the CRC appears to be somewhat lagging. Recently, a novel classification system for CRC, known as the consensus molecular subtypes (CMS), has been introduced. This system is based on multiple characteristics, including specific mutations [5]. Next-generation sequencing (NGS) enables comprehensive genome sequencing and elucidates the mutational signatures within cancer cells. Additionally, NGS facilitates microbiome analysis, which is emerging as a novel biomarker in CRC. Key areas of interest include mutations in the NRAS, KRAS, and BRAF genes. NGS methods have proven reliable in identifying microsatellite instability (MSI), a critical factor in prognosis and treatment. The integration of NGS with multi-omics studies presents new opportunities in epigenetics and transcriptomics. Utilizing NGS to consider the microbiome as a biomarker and to investigate the underlying pro-oncogenic mechanisms of various CRC-associated bacteria is promising. Moreover, metagenomics, metatranscriptomics, and meta-omics approaches are necessary to understand the mechanisms linking the microbiome to carcinogenesis [25,87,88,89,90,91,92]. Genetic aberrations and microbiome imbalances may be associated with metabolomic alterations in CRC. Consequently, there is substantial potential for employing multiple methods to comprehensively evaluate the underlying mechanisms and pathways that lead to specific pathological changes.

The human genome consists of 22,000 genes. NGS studies enable proper diagnosis in only about 30–40% of cases. Additionally, the functions of most known human genes are unknown and not associated with any human disease [93]. Identifying new genetic factors is of great importance to science: It deepens our understanding of the etiology and pathomechanisms of metabolic diseases and enhances our comprehension of human developmental biology. It should be noted that high-throughput genetic studies allow the discovery of pathogenic changes in the genome that are not always associated with the patient’s phenotype, making it difficult to predict their impact and establish a clear correlation with observed clinical symptoms. Integrating metabolomic, lipidomic, and proteomic data with genome sequencing data is a promising systematic approach to identifying disease-causing variants. Genomics, metabolomics, lipidomics, peptidomics, and proteomics are currently the most dynamically developing fields, crucial in modern approaches to early diagnosis and population profiling to obtain highly specific and personalized data. The search for new biomarkers, including lipid biomarkers, in conjunction with NGS data will enable the association of genes with the pathomechanism of disease onset and progression. Recent publications in lipidomics research in relation to NGS data support the validity of this approach [93,94,95,96].

## 5. Conclusions

The comprehensive exploration of lipidomic changes associated with CRC through blood and tissue sample analyses offers valuable insights into its pathogenesis. While tissue studies provide a detailed understanding of lipid composition in the tumor microenvironment, blood sample studies reflect systemic changes potentially serving as diagnostic or prognostic biomarkers. Despite their informative nature, both approaches face challenges, including sample availability and methodological heterogeneity.

The comparison between metastatic and non-metastatic CRC sheds light on distinct lipidomic alterations associated with disease progression. Tissue and blood sample analyses reveal significant differences in lipid composition between these two states. Notably, dysregulation of the AA/EPA ratio may be considered as a promising biomarker.

This review has several limitations, some of which are related to the limited publication period of the evaluated manuscripts. The data presented in the studies are often highly heterogeneous in terms of patient populations, age stratification, methodologies employed, and cancer stages. Unfortunately, the data concerning young-onset CRC or familial syndromes within the reviewed material are too scarce to adequately summarize and analyze.

In conclusion, CRC is characterized by multiple alterations in lipid metabolism. Various lipid species have been proposed as potential diagnostic markers, often in sets. However, these markers have yet to find practical application in daily clinical practice. Moving forward, it is essential to standardize analytical methods to ensure reliable results. Large-scale cohort studies are necessary to establish correlations and determine the diagnostic strength of proposed biomarkers.

## Figures and Tables

**Table 3:** Testing materials comparison.

Testing Material	Advantages	Drawbacks
Blood samples	Quick, easy, and non-invasive sampling; Screening and early diagnostic possibilities; Whole lipidome analysis	Lipidome changes may be due to other diseases; Blood is not tissue-specific to CRC
Tissue samples	The biological sample reflects the metabolomic alterations in the specific cancer tissue; Sample resistant to disturbing factors such as concomitant diseases; Potentially new prognostic markers based on lipidomic profiling	Taking samples requires an invasive procedure (endoscopy or surgery)
Cell culture assay	Allows for metabolic pathways exploration; Possible intervention and testing of drugs; Controlled study environment	In vitro studies do not fully reflect the in vivo metabolism; Time-consuming; Mostly restrained to commercially available cell lines
Preclinical animal models	Both blood and tumor testing; Possible intervention and drug testing in vivo; Controlled study environment; Allows for complete lipidomic analysis and the evaluation of specific alterations between blood and cancer tissue	Animal models may not be representative of human metabolism
